# Bilateral Naevus of Ito and Ota With Palatal Involvement

**DOI:** 10.7759/cureus.57004

**Published:** 2024-03-26

**Authors:** Niranjana S Pillai, Yash Buccha, Rahul S Nair, Rohit Kothari, Nishtha Malik

**Affiliations:** 1 Department of Dermatology, Venereology and Leprosy, Dr. D. Y. Patil Medical College, Hospital & Research Centre, Dr. D. Y. Patil Vidyapeeth (Deemed to be University), Pune, IND; 2 Department of Orthopaedic Surgery, Holy Cross Hospital, Kollam, IND

**Keywords:** acquired nevi, congenital melanocytic nevi, hori naevus, naevus of ito, naevus of ota

## Abstract

Naevus of Ito and naevus of Ota are benign dermal melanocytoses with similar pathogenic mechanisms of failure in the melanocyte migration to typical locations within the basal layer from neural crest cells and differ in distribution. Bilateral and oral mucosal involvement of naevus of Ota can occur but is infrequent. Naevus of Ito is seldom associated with naevus of Ota and extracutaneous manifestations. A review of the English literature showed 14 cases of naevus of Ota with palatal involvement. None showed bilateral involvement of both naevi with oral involvement. Here we report the case of bilateral naevus of Ito and bilateral naevus of Ota with palatal involvement. A 32-year-old male came to us with naevus of Ito on both sides of his back and naevus of Ota on both sides of his face involving the sclera of both eyes with a bluish lesion along the midline of the hard palate.

## Introduction

The naevus of Ota and the naevus of Ito are types of dermal melanocytoses with differences in the territory of distribution. Naevus of Ota described by Masao Ota in 1939 are unilateral bluish-brown patches along the distribution of the first and second branches of the trigeminal nerve involving the sclera and rarely the palate since birth with female preponderance [1]. In 1954, Minor Ota described naevus of Ito as a unilateral bluish-grey macular discoloration involving the area innervated by the posterior supraclavicular and lateral cutaneous brachial nerves supplying the deltoid, supraclavicular and scapular regions [2,3]. Typically, naevus of Ota and naevus of Ito occur unilaterally.

Recently, we happened to see a very rare association of bilateral naevus of Ito along the distribution of thoracodorsal nerve and bilateral naevus of Ota with palatal involvement.

## Case presentation

A 32-year-old male, born out of a non-consanguineous marriage, presented to us with dark-colored lesions on his face, eyes, mouth, and back since childhood. Until 12 years of age, the lesions were asymptomatic, progressively increased in size, and have remained static ever since. On inquiry, no family history of similar lesions was present.

**Figure 1:**
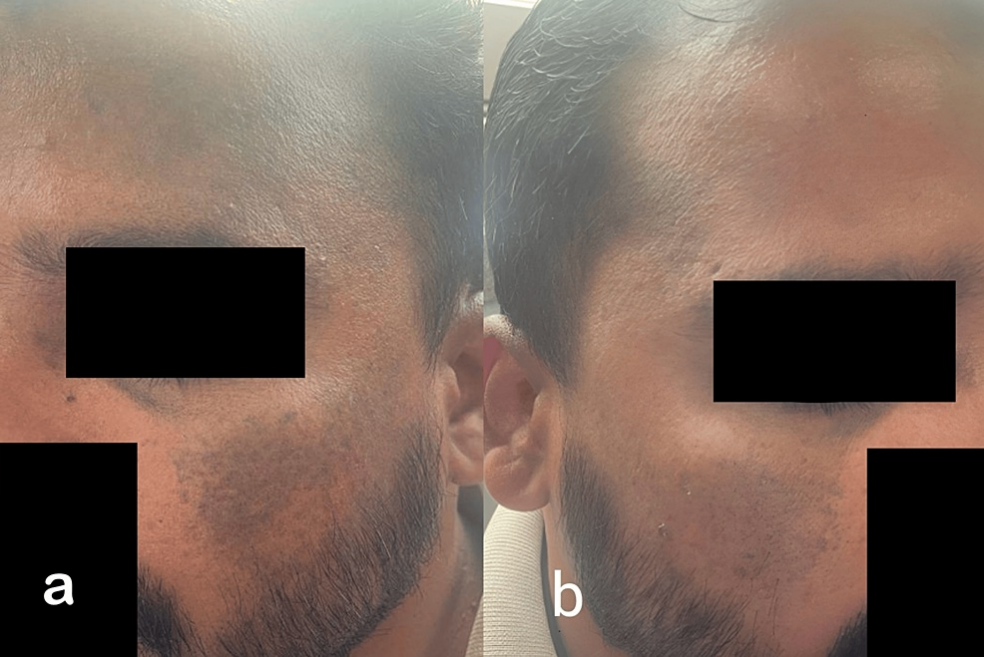
Naevus of Ota a, b: Bluish-grey colored macules to patches present over the periorbital area, cheeks extending up to the temples and forehead on the left and right side respectively

**Figure 2:**
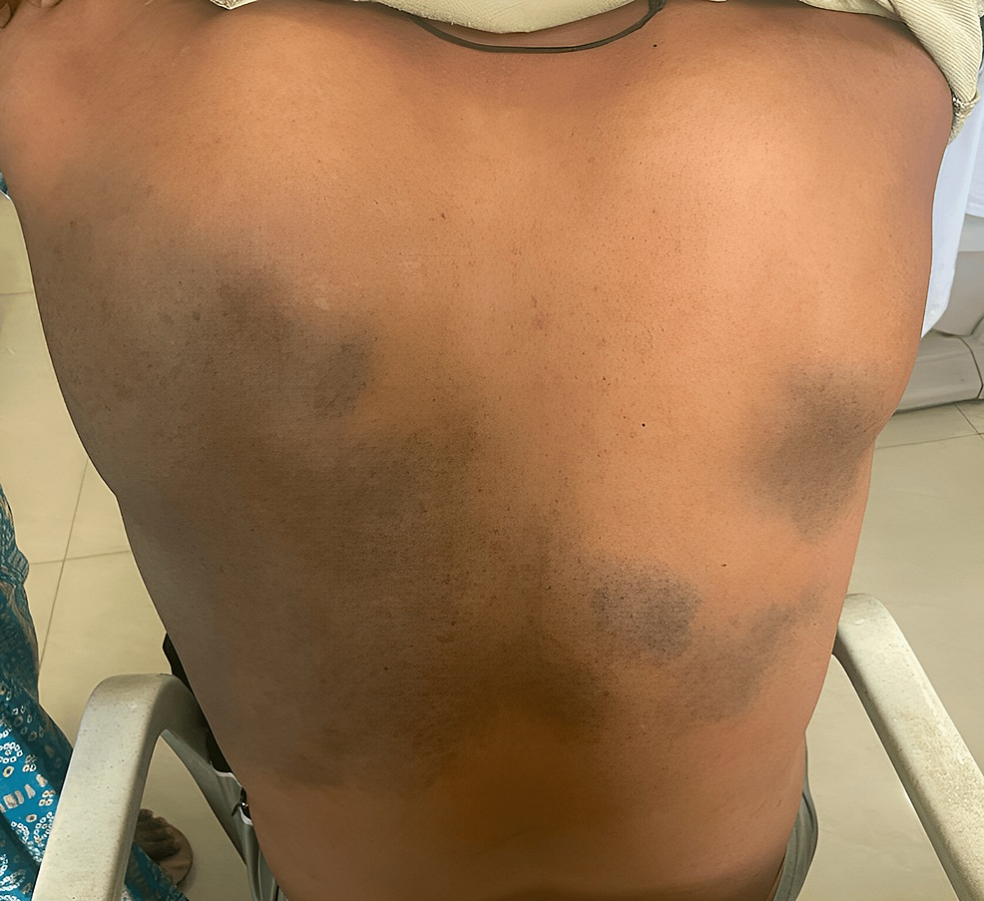
Naevus of Ito Bluish-grey speckled macules to patches on bilateral lumbar, infrascapular, and left scapular area

On cutaneous examination, multiple ill-defined bluish-grey macules to patches were present asymmetrically over the bilateral periorbital regions, symmetrically over the bilateral malar area extending up to the temples and the forehead on the left side and temple region on the right (Figure 1). In addition, the patient had multiple bluish-grey speckled macules to patches on the bilateral lumbar, infrascapular, and left scapular area (Figure 2).

**Figure 3:**
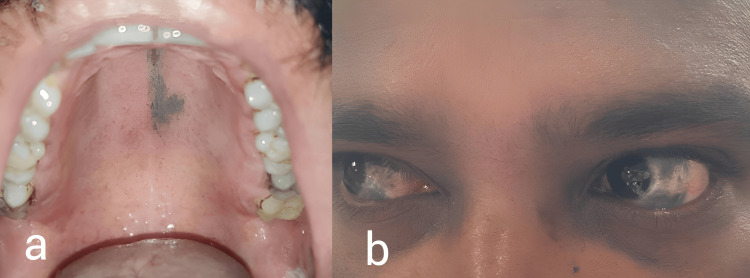
Naevus of Ota with mucosal involvement a: Deep-blue irregular mottled patch on the hard palate; b: Bluish-grey discoloration of bilateral sclera

On examining the oral mucosa, a deep blue irregular mottled patch was noted on the hard palate (Figure 3a), and bluish-grey pigmentation of the sclera was noted (Figure 3b). On slit lamp examination, marked patchy bluish-grey episcleral pigmentation was present in all quadrants of bilateral eyes. Visual acuity testing, intraocular pressure measurement, and dilated fundus examination showed no abnormality. Otoscopy revealed a normal tympanic membrane.

**Figure 4:**
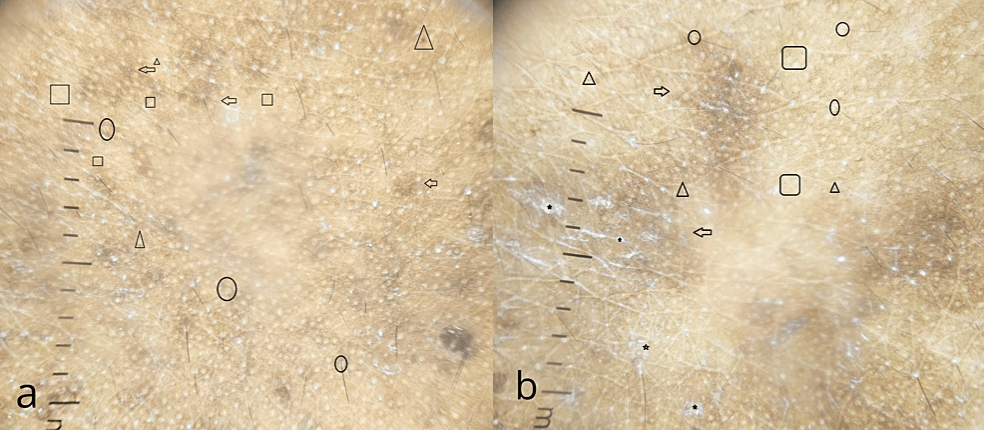
Dermoscopic images a: Naevus of Ota showing brownish gray structureless areas in patchy distribution (arrow) with scattered brown dots (triangle), terminal hair with perifollicular hyperpigmentation (circle), and four dot clods (square); b: Naevus of Ito showing brownish gray structureless areas in patchy distribution (arrow) with scattered brown dots (triangle), scaling (star), terminal hair with perifollicular hyperpigmentation (circle) and four dot clods (square)

Dermoscopic examination of the naevus of Ota showed brownish-gray structureless areas in patchy distribution with scattered brown dots, terminal hair with perifollicular hypopigmentation, and four dot clods (Figure 4a) while the naevus of Ito showed brownish-gray structureless areas in patchy distribution with scattered brown dots, scaling, terminal hair with perifollicular hypopigmentation and four dot clods (Figure 4b).

**Figure 5:**
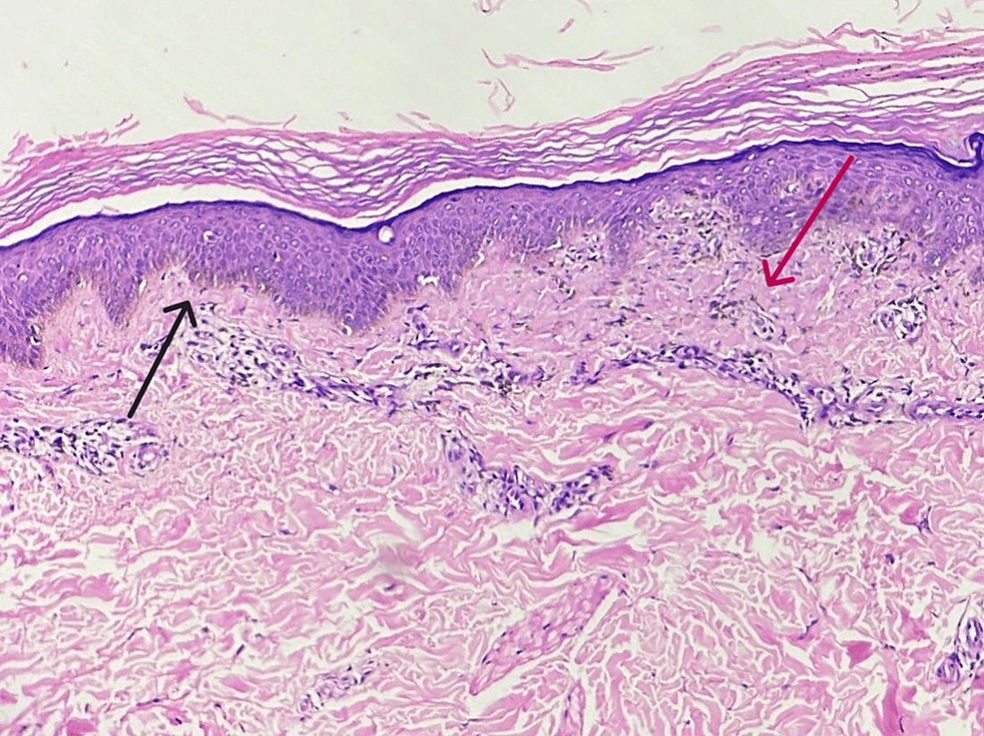
Histopathological image The black arrow shows increased basal pigmentation and the pink arrow shows scattered long slender, pigmented dendritic dermal melanocytes

Histopathologic examination of hyperpigmented macules over the back showed heavily pigmented long, slender dendritic dermal melanocytes scattered in the upper, middle, and lower dermis with increased basal layer pigmentation (Figure 5). The patient refused to consent to a biopsy of the cheek. 

**Figure 6:**
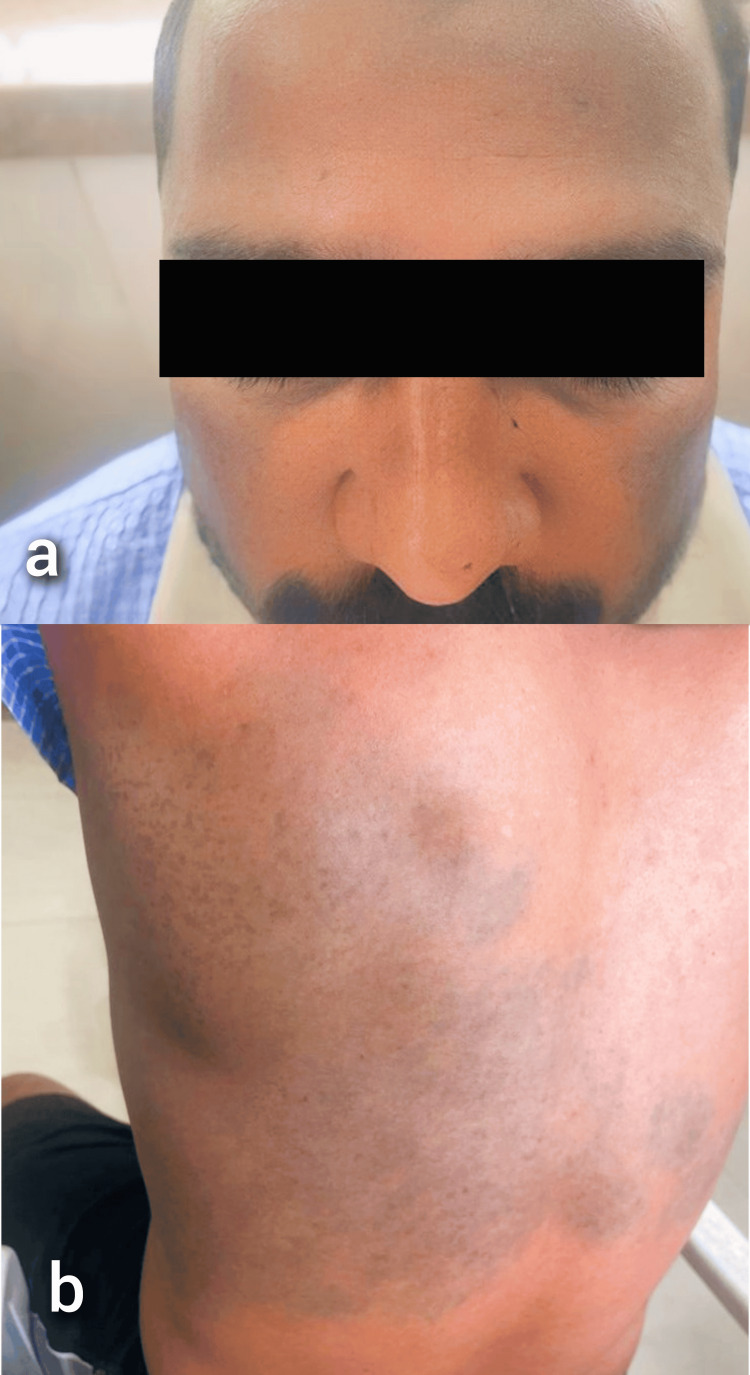
Post-treatment images a: Showing Naevus of Ota; b: Showing Naevus of Ito

The individual received treatment using a Q-switched Nd:YAG laser. A total of six sessions were conducted, with a two-month interval between each session. The treatment was performed in two passes. As a result, there was a significant improvement in appearance (Figure 6).

## Discussion

Dermal dendritic melanocytic proliferations, a clinical spectrum of congenital and acquired melanocytic lesions, historically fall into three categories: blue naevi, malignant blue naevi, and dermal melanocytoses [4].

Dermal melanocytosis is commonly seen in the Asian female population and is distinguished by the differences in the course and distribution of lesions. Although the exact etiology is unknown, the following theories have been proposed: failure of migration of melanocytes to the basal layer of the epidermis, melanoblasts migrate to skin and uvea during embryogenesis, mutation of GNAQ (G-coupled protein) constitutively activating the G-coupled protein, and previous exposure to radiation, radiotherapy or hormonal factors [5]. The degree of involvement determines the color of the lesions. Due to the Tyndall effect, the deeper lesions seem blue, while the more superficial lesions have a slate grey hue [6]. Treating dermal melanocytoses can be challenging, although the literature shows satisfactory results with pigment-specific lasers.

Fractional Erbium:YAG (2940 nm) and Q-switched Nd:YAG (1064 nm) lasers are found to be efficacious in the treatment of naevi. With water as the target chromophore, the Erbium: YAG laser ablates tissue to treat the hyperpigmented parts of naevi. Using thermal damage to destroy the lesion by denuding the epidermis, the Erbium:YAG laser eliminates superficial pigmented lesions. Microscopic vaporization channels can extend up to 3-4 mm into the dermis as a result of fractional Erbium:YAG causing minor thermal damage zones called microthermal treatment zones (MTZs). Desquamation of the epidermis of each MTZ removes basal layer pigment and a tiny quantity of papillary dermal detritus. By modifying the parameters, the operator can decide how deep these MTZs are. The depth of ablation can be extended much deeper than with standard ablative resurfacing procedures because remodelling begins in the spared tissue between the ablation columns. A Q-switched Nd: YAG laser can cure naevi hyperpigmentation by targeting the body's natural melanin pigment. A pigment-selective laser, the Q-switched Nd:YAG laser destroys melanin in the dermis or epidermis while leaving the skin unharmed. Melanin absorbs light more strongly than other pigments because of the pico- or nanosecond pulses it emits. Therefore, the pigment is the only target of the energy, which means it doesn't affect the surrounding tissues in any way. Both cutaneous melanophages and melanocytes were destroyed by the Q-switched Nd:YAG 1064 nm laser. Results from studies using both dermal and epidermal components to treat pigmented lesions were promising. Because it lessens the likelihood of epidermal injury, it is also an excellent choice for treating dark-pigmented (Fitzpatrick skin types IV-VI) patients [7]. Chances of recurrence are present from six months to two years post-treatment if precautions aren't taken post-procedure for the said time period. These precautions include sun protection, and avoidance of unknown cosmetic products both of which may cause skin damage and hence recurrence.

In this case, the patient has facial dermal melanocytoses - naevus of Ota with a close differential diagnosis of Hori’s naevus (acquired, bilateral naevus of Ota-like macules). Differences between the naevus of Ota, Hori’s naevus, the naevus of Ito, and the present case have been shown in Table 1 [8-11].

**Table 1:** Difference between the naevus of Ota, Hori’s naevus, naevus of Ito, and the present case

	Naevus of Ota	Hori’s naevus	Naevus of Ito	Present case
Onset	Congenital	Acquired	Congenital	Congenital
Color of lesion	Blue-grey to slate brown	Blue-brown or slate-grey	Blue-grey to slate brown	Bluish-grey
Symmetry	Asymmetrical	Symmetrical	Asymmetrical	Symmetrical - malar area, Asymmetrical - forehead
Site	Unilateral, Ophthalmic and maxillary branches of the trigeminal nerve - forehead, temples, periorbital area, nose, and cheek	Bilateral forehead, temples, eyelids, and nose	Unilateral posterior supraclavicular and lateral cutaneous brachial nerves - deltoid, supraclavicular, and scapular regions	Bilateral forehead, temples, periorbital area, and cheeks; Thoracodorsal nerve - bilateral lumbar, infrascapular, and left scapular area
Mucosal involvement	Sclera, conjunctiva, palate, buccal mucosa, nasal mucosa	Absent	Absent	Sclera and palate
Dermoscopy	Brownish-gray structureless areas with scattered brown dots and white dots in a four-dots-clod arrangement	Blue-brown or gray with speckled homogenous pattern	Bluish to slate grey homogeneous pigmentation	Face - brownish-gray structureless areas in patchy distribution with scattered brown dots, terminal hair with perifollicular hypopigmentation, and four dot clods. Back - brownish gray structureless areas in patchy distribution with scattered brown dots, scaling, terminal hair with perifollicular hypopigmentation and four dot clods
Histopathology	Numerous elongated, slender, pigment-bearing dendritic dermal melanocytes are distributed throughout the dermis and dispersed in between the collagen fibers of the dermis with their long axis along the collagen fibers	Elongated, slender, heavily pigmented dendritic dermal melanocytes scattered in the upper dermis and more frequently in the perivascular area	Numerous elongated, slender, pigment-bearing dendritic dermal melanocytes are scattered throughout the dermis	Numerous elongated, slender, pigmented dendritic dermal melanocytes are scattered throughout the upper, middle, and lower dermis with increased basal pigmentation

## Conclusions

Our case had parallels to naevus of Ota in terms of the timing of emergence, location of the lesions, and involvement of mucosal areas such as the sclera and hard palate. However, it differed in terms of symmetry. Consequently, the likely diagnosis is a bilateral naevus of Ota instead of a Hori naevus. We want to emphasize the rarity of the occurrence of this triple association between Bilateral naevus of Ota and Ito with palatal involvement. The psychological consequences of these lesions are substantial, and immediate management with laser therapy would considerably ease the distress felt by the patient.
